# Spatio-Temporal Environmental Correlation and Population Variability in Simple Metacommunities

**DOI:** 10.1371/journal.pone.0072325

**Published:** 2013-08-30

**Authors:** Lasse Ruokolainen

**Affiliations:** Department of Biosciences, Helsinki University, Helsinki, Finland; McGill University, Canada

## Abstract

Natural populations experience environmental conditions that vary across space and over time. This variation is often correlated between localities depending on the geographical separation between them, and different species can respond to local environmental fluctuations similarly or differently, depending on their adaptation. How this emerging structure in environmental correlation (between-patches and between-species) affects spatial community dynamics is an open question. This paper aims at a general understanding of the interactions between the environmental correlation structure and population dynamics in spatial networks of local communities (metacommunities), by studying simple two-patch, two-species systems. Three different pairs of interspecific interactions are considered: competition, consumer–resource interaction, and host–parasitoid interaction. While the results paint a relatively complex picture of the effect of environmental correlation, the interaction between environmental forcing, dispersal, and local interactions can be understood via two mechanisms. While increasing between-patch environmental correlation couples immigration and local densities (destabilising effect), the coupling between local populations under increased between-species environmental correlation can either amplify or dampen population fluctuations, depending on the patterns in density dependence. This work provides a unifying framework for modelling stochastic metacommunities, and forms a foundation for a better understanding of population responses to environmental fluctuations in natural systems.

## Introduction

Natural populations experience fluctuating environmental conditions (such as temperature and precipitation). This variation occurs over time in local habitats, but there is also variation in conditions across space. Temporal variation is often positively autocorrelated, i.e., consecutive observations tend to be more similar to each other than those separated by longer time lags [1]. Similarly, more closely located areas tend to be more similar in their local environmental conditions, implying positive spatial autocorrelation. That is, environmental variation tends to be autocorrelated both over time and across space, which is expected to have both ecological and evolutionary consequences on biological systems [2]. Understanding how spatio-temporal patterns in environmental fluctuations, and those in species-specific responses to these fluctuations affect variability in population densities is an important challenge, when planning sustainable conservation and exploitation of natural populations. This is reflected in the recent interest in studying stochastic metacommunities both theoretically and empirically [3]–[7].

When considering the dynamics of populations and communities in a spatial context, the influence of local environmental fluctuations can be extended to other localities via individual dispersal between habitats. A common pattern is that increased migration between habitats stabilises local population dynamics that are not fully synchronised [3], [8], [9]. However, when the correlation between local environmental conditions increases (i.e., localities become more similar in their environments), the associated increase in population synchrony increases the extinction risk of the entire metapopulation [3], [6], [10], [11]. Increasing local temporal autocorrelation in the environment can either increase or decrease population variability, depending on the strength of population density dependence, between-species interaction strength, and local community structure [12]–[16].

In general, species can differ in their responses to environmental fluctuations, e.g., depending on the similarity of their environmental tolerances [17]–[19]. When two species that are differently adapted to a common environmental variable come to interact, the arising between-species environmental correlation (*ρ_S_*) – *ρ_S_* = 1 means that responses are identical,*ρ_S_* = 0 means responses are independent, and *ρ_S_* = –1 indicates completely opposite responses – can affect population variability in closed communities [2], [14], [15], [20]. In simple competitive communities increasing *ρ_S_* dampens undercompensating population fluctuations, whereas overcompensating populations tend increase in variability [14], [15], [21]. In multi-trophic food webs increased *ρ_S_* can be associated with lower population variability [22], as well as increased food web persistence [23].

In the metacommunity context, where local communities are coupled together via dispersal of one or several species [24], the relative influence of between-patch (*ρ_E_*) and between-species environmental correlation (*ρ_S_*) on population variability remains unknown. This paper aims at filling this gap in ecological theory. Here I investigate simple two-patch, two-species stochastic systems and ask how patterns in local population variability (*CV* of population density) are affected by independently varying *ρ_E_* and *ρ_S_*. To gain a general understanding of how the environmental correlation structure might affect spatial community dynamics, I consider three different metacommunity types with different between-species interaction patterns: (1) competitive, (2) consumer–resource, and (3) host–parasitoid metacommunities. When local dynamics are stable (in the absence of environmental variation), community dynamics can be analytically approximated around the equilibrium point for each spatial system (analytical results are verified by stochastic simulations).

The results show that *ρ_E_* and *ρ_S_* interact in affecting population variability in simple spatially extended communities (i.e., metacommunnities). Patterns of population variability are mainly governed by two mechanisms: (1) the coupling between immigration and local densities, and (2) the coupling between local populations under forced synchronisation. The first mechanism explains why reduced *ρ_E_* is always associated with reduced population variability. The second mechanism explains why different communities can have opposite responses to increased *ρ_S_*. In competitive communities the effect of *ρ_S_* depends on the strength (or shape) of density-dependence, whereas in exploitative communities this depends on the importance of top-down versus bottom-up effects (here also related to patterns in density-dependence) affecting community dynamics. The effects of the interaction between different sources of environmental correlation (*ρ_E_* and *ρ_S_*) have not been fully appreciated before. Thus, the present paper deepens our understanding of spatio-temporal variation in species communities under stochastic environmental conditions, as well as provides a framework for future studies, e.g., on more complicated metacommunities, with different spatial structures and food web topologies.

## Methods

### Spatial community dynamics

A general model for two-patch, two-species population dynamics in discrete time is given as [3], [25]:
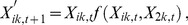
(1a)

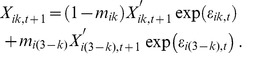
(1b)


In this model eqn. (1a) describes local population dynamics and eqn. (1b) is the dispersal process. *X_ik_*_,*t*_ denotes the density of population *i* (*i* ∈1,2) in patch *k* (*k* ∈1,2) at time *t*. Function *f* stands for per capita growth rate, which depends on the density of species 1 and 2 in that patch. Before dispersal, populations are forced by species- and patch-specific environmental stochasticity *ε_ik_*_,*t*_, which can be correlated both between species and between patches. Dispersal redistributes individuals between the patches such that a fraction 1 – *m_ik_* remains in the natal patch (*m* = 0 no one moves, *m* = 1 everyone moves), whereas a proportion *m_i_*_(3 – *k*)_ of individuals born in patch *k* move to patch 3– *k* [3], [25], [26]. While the order of events is unlikely to affect results [27], local dynamics are here assumed to precede dispersal, following earlier work [27], [28].

When patches are identical, equilibrium population sizes become independent of dispersal propensity *m_ik_* (dispersal does not need to be symmetric between patches, as the dispersal process is density-independent, and patches are identical). This simplifying assumption facilitates analytical treatment of eqn. (1).

**Figure 1:**
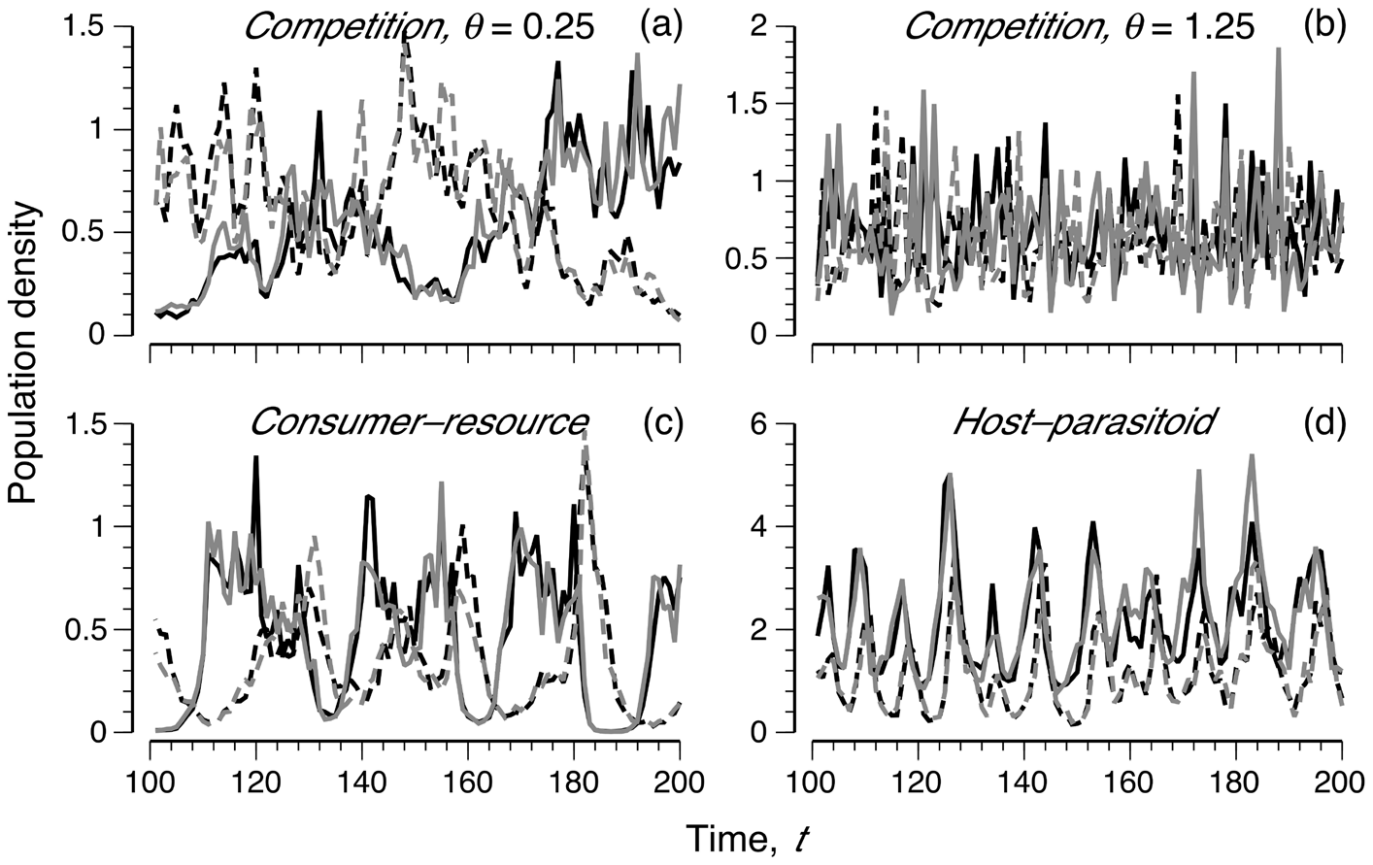
Examples of stochastic time series for two-species (species 1 solid, species 2 dashed), two-patch metacommunities (black lines for patch 1 and gray lines for patch 2). Local communities consist of either competitive communities with (a) undercompensating or (b) overcompensating populations, a (c) consumer–resource system (resource solid, consumer dashed), or a (d) host–parasitoid system (host solid, parasitoid dashed). Parameters: (a) *r* = 1.5, *θ* = 0.25, α = 0.5; (b) *r* = 1.5, *θ* = 1.25, α = 0.5; (c) *r* = 1, *K* = 1, *a* = 2, *R*_0_ = 1.25, *e* = 0.5, *d* = 0.25; (d) *r* = 2, *q* = 0.5, *b* = 0.5. In all cases *m_ik_*  =  *m* = 0.25, and environmental variation is white noise (zero mean and variance σ^2^ = 0.01) affecting population per capita growth rates, independently between patches and species (i.e., *ρ_E_*  =  *ρ_S_* = 0).

The main interest here is to analyse how different environmental correlation structures affect population variability. For demonstration, three different alternatives for local dynamics (eqn. 1a) are considered. First, a multi-species theta-Ricker model of between-species competition:
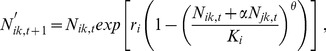
(2)where *N_ik_*_,*t*_ is the population density of species *i* in patch *k* at time *t* and *r_i_* is it's intrinsic rate of increase and *K_i_* is it's carrying capacity. Parameter *α* defines the strength of interspecific competition, and *θ* specifies the shape of density dependence [29]. For simplicity, parameters are set equal for both species (and patches): *r* = 1.5, *K* = 1, and *α* = 0.5. For parameter *θ* two cases are considered: undercompensating population dynamics with *θ* = 0.25 (fig. 1a), and overcompensating dynamics with *θ* = 1.25 (fig. 1b) (*θ*>1 is required for overcompensation, as well as *α* < *θr* –1). Equilibrium densities in this system are N_i_*  =  *K*/(1+*α*). This equilibrium is stable, given the value of *α*, if |1– *θr*|<1.

A second example considers a consumer-resource model with a saturating functional response [30]:
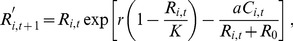
(3a)

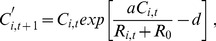
(3b)where *R* and *C* indicate resource and consumer populations, respectively. Parameters *r* and *K* are as in eqn. (2), *a* is the maximum intake rate of the consumer, *R*_0_ is the half-saturation constant, *e* is the consumers conversion efficiency, and *d* is consumer mortality. Again, parameters are chosen such that long-term population dynamics are stable in the absence of environmental variation: *r* = 1, *K* = 1, *a* = 2, *R*_0_ = 1.25, *e* = 0.5, *d* = 0.25, with equilibrium densities: R_i_*  =  *R*_0_ *d*/(*ae – d*) and C_i_*  =  *rR*_0_[*Kae* ^2^ – *de*(*R*_0_ + *K*)]/*K*(*d* – *ae*)^2^ (see fig. 1c for an example time series for consumer–resource dynamics). For the consumer to persist, it is required that *d* < *Kae*/(*B*_0_ + *K*). A general requirement for the stability of this equilibrium is that *d*/*e* > *a*(*B*_0_ – *K*)/(*B*_0_ + *K*) [31].

The third example considers host-parasitoid dynamics (using the so-called “negative-binomial” model [32]):
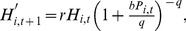
(4a)

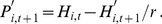
(4b)Here *H* and *P* stand for the host and the parasitoid, respectively. When there are no parasitoids, hosts grow exponentially with rate *r*. Parasitoids attack hosts with rate *b*, and the parasitoid attacks are distributed according to a negative binomial distribution among hosts, with shape parameter *q.* Parasitoid density depends on the proportion of parasitised hosts. Stable coexistence of *H* and *P* requires that *r*>1 and *q*<1 [3]. Model parameters are set to *r* = 2, *b* = 0.5, and *q* = 0.5, which results in a stable equilibrium with population densities of H_i_*  =  *qr*(*r* ^1/*q*^ –1)/*b*(*r* –1) and P_i_*  =  *q*(*r* ^1/*q*^ –1)/*b* [32] (an example time series for host–parasitoid dynamics is given in fig. 1d). This system is globally stable only if the equilibrium is stable (i.e., there is no stable limit cycle).

### Linear analysis

For simplicity, environmental variation *ε_ik_* is assumed to be serially uncorrelated ‘white’ noise, with a covariance matrix **C** (see Appendix S1), describing the correlation in between-patch environmental fluctuations (between-patch environmental correlation, *ρ_E_*) and how similarly the two species react to these fluctuations (between-species environmental correlation, *ρ_S_*). When linearised around the equilibrium point, local population dynamics in each system can be described by the Jacobian matrix **J** (Appendix S1), while dynamics in the global metacommunity are governed by matrix **B** = **MJ**, where **M** is a dispersal matrix that describes the way individuals move between the local communities (Appendix S1). Given that equilibrium dynamics are stable in the absence of stochasticity (as required above), a linear approximation of the population variance-covariance matrix **V** can be obtained as follows, assuming white noise [12]:
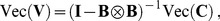
(5)where Vec(**V**) is the vectorised variance-covariance matrix. The ⊗ symbol indicates the Kronecker tensor product (producing all possible combinations between the elements in two matrices), **I** is an identity matrix (ones on the main diagonal and zeros elsewhere), and Vec(**C**) is the vectorised environmental covariance matrix. The diagonal elements of **V** contain population variances *V_ii_*, while the off-diagonal elements are between-population covariances *V_ij_*.

While eqn. (5) is a useful tool for obtaining population variances (and covariances), it is not particularly useful for doing detailed analysis of the relationship between biological parameters and environmental characteristics [15]. This can be in principle achieved via transforming the system described by **B** to the coordinates along the eigenvectors of **B** and back-transforming the resulting variances to gain variances in population densities [14], [15], [17]. For the first scenario, with interspecific competition (eqn. 2), population variance is readily obtained using this method (Appendix S1):
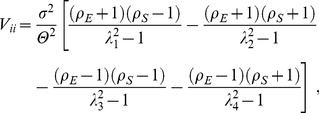
(6)where *σ* ^2^ is the environmental variance, *λ_i_* are the eigenvalues of **B** (Appendix S1), and Θ is the order of **B** (here Θ  =  4). Population variability is then found as *CV*  =  (α + 1)(*V_ii_*)^1/2^/*K*. From eqn. (6) it can be seen that the relative magnitude of *λ_i_* affects the way *ρ_E_* and *ρ_S_* impact on population variance. However, the patterns in which the eigenvalues interact with environmental correlations are far more complex than those in isolated systems [14], [15]. Eqn. (6) can be expanded to reveal the influence of model parameters on the relative importance of the interaction between different sources of environmental correlation (*ρ_E_ρ_S_*; Appendix S1, Figure S1, Text S1). This variance component becomes more important with increasing *θ* and *r*, less important with increasing α, and less (more) important with increasing *m* (when *m_ik_*  =  *m*) when intrinsic dynamics are undercompensatory (overcompensatory).

Eqn, (6) gives the variance of local population fluctuations. The variance of global population variance, i.e., the variance of summed population densities (across patches) can be found by summing across the population variance-covariance matrix **V** for each species:
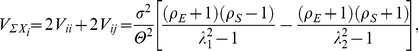
(7)where *V_ii_* is local population variance (eqn. 6) and *V_ij_* is between-population covariance. Unlike *V_ii_*, *V_ΣX_* is independent of the magnitude of dispersal, as the eigenvalues *λ*_1_ and *λ*_2_ are independent of *m_ik_*. The remaining analysis concentrates on *V_ii_* , which are used to calculate the population coefficient of variation, *CV_ik_*  =  (*V_ik_*)^½^/X_ik_* (where X_ik_* is the equilibrium density of population *i* in patch *k* ), a commonly used statistic for measuring population variability [33]–[35].

For systems in eqns. (3, 4) with consumer–resource and host–parasitoid dynamics, respectively, simple analytical expressions for population variances cannot be obtained. This is because the eigenvectors of the metacommunity Jacobian (**B**) depend on model parameters, which leads to the covariance matrix for dynamics along the eigenvectors having non-zero off-diagonal elements. The interpretation of such values is not straightforward, as the variance along eigenvectors should be independent by definition [14]. Therefore the remaining analysis utilises eqn. (5) to generate numerical results for symmetric dispersal. Between-population synchrony (zero-lag cross-correlation) can be obtained from eqn. (5) as 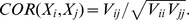


### Stochastic simulations

Depending on the amplitude of environmental stochasticity, analytical predictions do not necessarily match simulation results [36]. The reliability of analytical results was evaluated by simulating spatial community dynamics for *t_MAX_*  = 25000 time steps (population densities initiated at random densities between (0, 1]) for each parameter combination (the first 5000 time steps were discarded before further analysis). The data was then used to calculate mean population variability for each species as the coefficient of variation [*CV_i_*  =  Σ*_i_*(σ(*X_ik_*)/*μ*(*X_ik_*))/2]. Each parameter combination was replicated 100 times. The procedure for stochastic simulation of the model communities, particularly the generation of environmental variables *ε_ik_*, is described in detail in Text S1.

## Results

**Figure 2:**
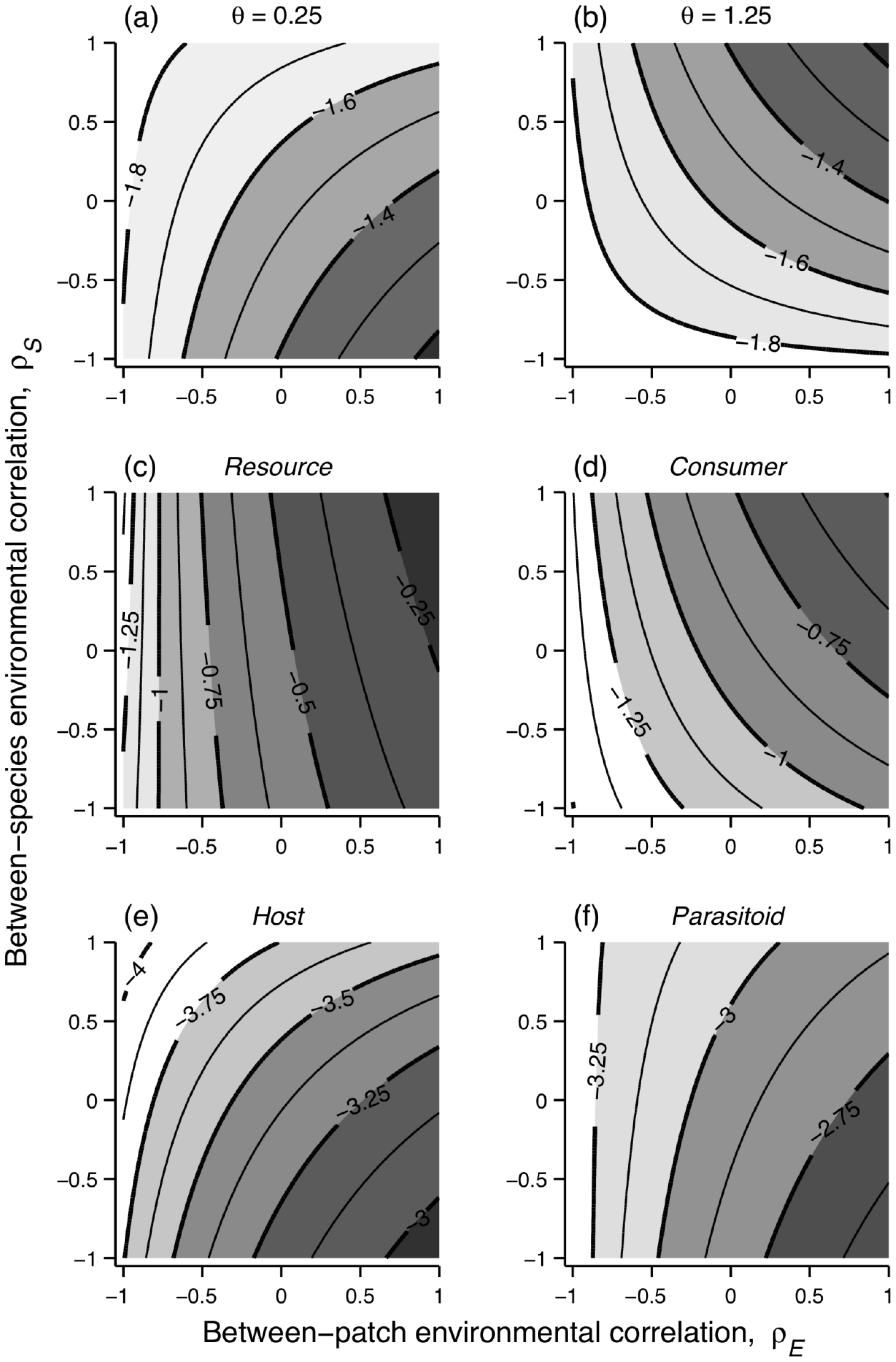
Population variability in simple two-species two-patch metacommunities depends on an interaction between the environmental correlation between patches (*ρ_E_*, describing the similarity in environmental conditions across space) and the local environmental correlation between species (*ρ_S_*, describing how similarly species respond to variation in local environmental conditions). This interaction further depends on the nature of the between-species interaction, as well as the ecological role of the focal population. In competitive communities (eqn. 2) species intrinsic dynamics are either (a) undercompensatory (*θ* = 0.25) or (b) overcompensatory (*θ* = 1.25). In exploitative communities (eqns. 3, 4) the interaction involves either (c, d) consumer–resource dynamics, or (e, f) host–parasitoid dynamics. The contours represent the logarithm of (local) population *CV*. Results are based on an intermediate level of symmetric dispersal for both species, *m_ik_*  =  *m* = 0.25 (note that the effect of varying *m* is symmetrical around 0.5). Environmental variation is serially uncorrelated white noise, with zero mean and variance σ^2^ = 0.01.

A general pattern common to all scenarios examined here is that decreasing between-patch environmental correlation (*ρ_E_*) decreases (local) population variability. This happens because increasingly asynchronous population fluctuations between patches become dampened by the act of dispersal; individuals dispersing from a large population to a small population bring both populations closer to their long-term means [9]. While varying *ρ_E_* leads to an intuitive and expected result in all cases, the way *ρ_E_* and the between-species environmental correlation (*ρ_S_*) interact in driving population variability is more complicated. The interaction between *ρ_E_* and *ρ_S_* depends on the nature of local between-species interactions, as well as on the ecological role of the focal population (fig. 2). This namely boils down to whether forced synchronisation is amplified or dampened by species interactions.

### Symmetric dispersal

In competitive metacommunities increasing *ρ_S_* reduces the variability of populations with undercompensating dynamics (fig. 2a), and increases variability in association with overcompensating dynamics (fig. 2b). In addition, the effect of increasing *ρ_E_* decreases with increasing (decreasing) *ρ_S_* for undercompensating (overcompensating) populations. These patterns arise because asynchronous local dynamics are amplified by undercompensating population responses to perturbations, whereas overcompensation amplifies synchronous dynamics [12], [36].

As with the competitive communities, the species interaction can either amplify or dampen population fluctuations in exploitative communities (*C–R* or *H–P*). In the consumer–resource system (top-down controlled) population variability increases with increasing *ρ_S_* (fig. 2c, d). Here unstable dynamics are driven by overconsumption [30], while in the absence of consumption resources approach their carrying capacities. Negatively correlated local environmental responses between species (*ρ_S_*<0) lead to situations where resources are favoured and consumers are pressed by environmental conditions, which reduces the predation pressure and dampens population fluctuations. Conversely, increasingly positive *ρ_S_* between the resource and the consumer promotes overconsumption, as the consumer is favoured when the resource is abundant. As in the *C–R* system, the dynamical stability of the host–parasitoid system is controlled by the coupling strength between the two species (bottom-up controlled); in a persistent system the parasitoid is able to control the exponential growth of the host. This means that increasingly positive *ρ_S_* will dampen population fluctuations by promoting this coupling, whereas increasingly negative *ρ_S_* will increase the size of population fluctuations by decoupling host and parasitoid densities (fig. 2e, f).

Patterns in population variability are in qualitative agreement with those in between-patch (within-species) population synchrony (Text S1), which means that patterns in global population variability reflect those in local population variability. Increasing *ρ_E_* is always synchronising. Instead, increasing *ρ_S_* desynchronises (synchronises) populations in undercompensating (overcompensating) competitive systems (Figure S2a, b). In consumer–resource systems increasing *ρ_S_* synchronises both resource and consumer dynamics between patches (Figure S2c, d), whereas host and parasitoid populations tend to become desynchronised (Figure S2e, f).

### Asymmetric dispersal

**Figure 3:**
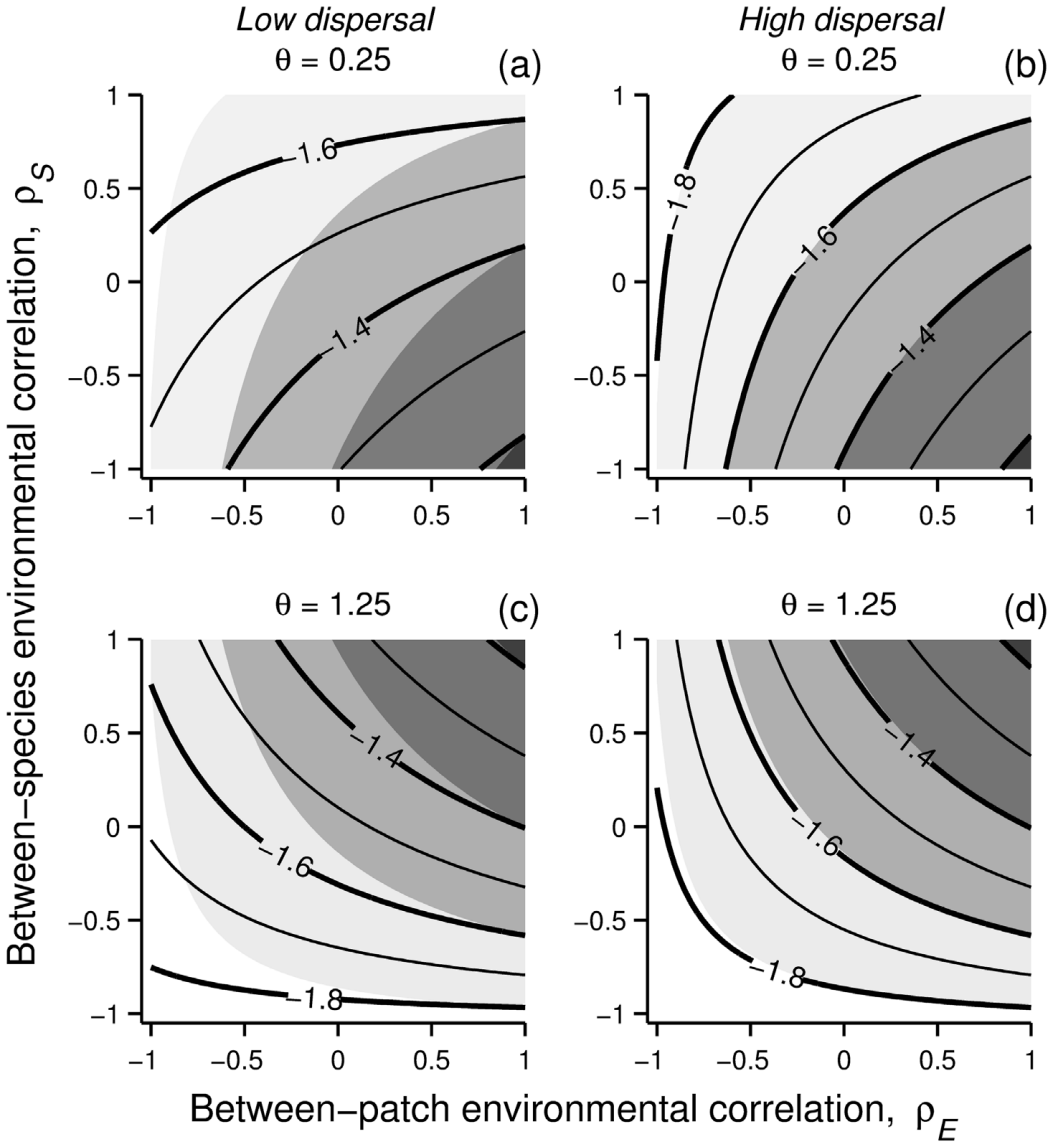
The effect of between-patch (*ρ_E_*) and between-species (*ρ_S_*) environmental correlation on population variability (log *CV*) in competitive metacommunities with asymmetric dispersal between species. The dispersal propensity is low (*m*_1*k*_ = 0.05) for one species (a, c) and relatively high (*m*_2*k*_ = 0.25) for the other (b, d). Populations have either undercompensatory (*θ* = 0.25; a, b) or overcompensatory (*θ* = 1.25; c, d) intrinsic dynamics. Contour lines represent (log) population *CV* under asymmetric dispersal, whereas the shading represents log *CV* under symmetric dispersal (with the same steps as the thick contour lines). Environmental variation is serially uncorrelated white noise, with zero mean and variance σ^2^ = 0.01.

**Figure 4:**
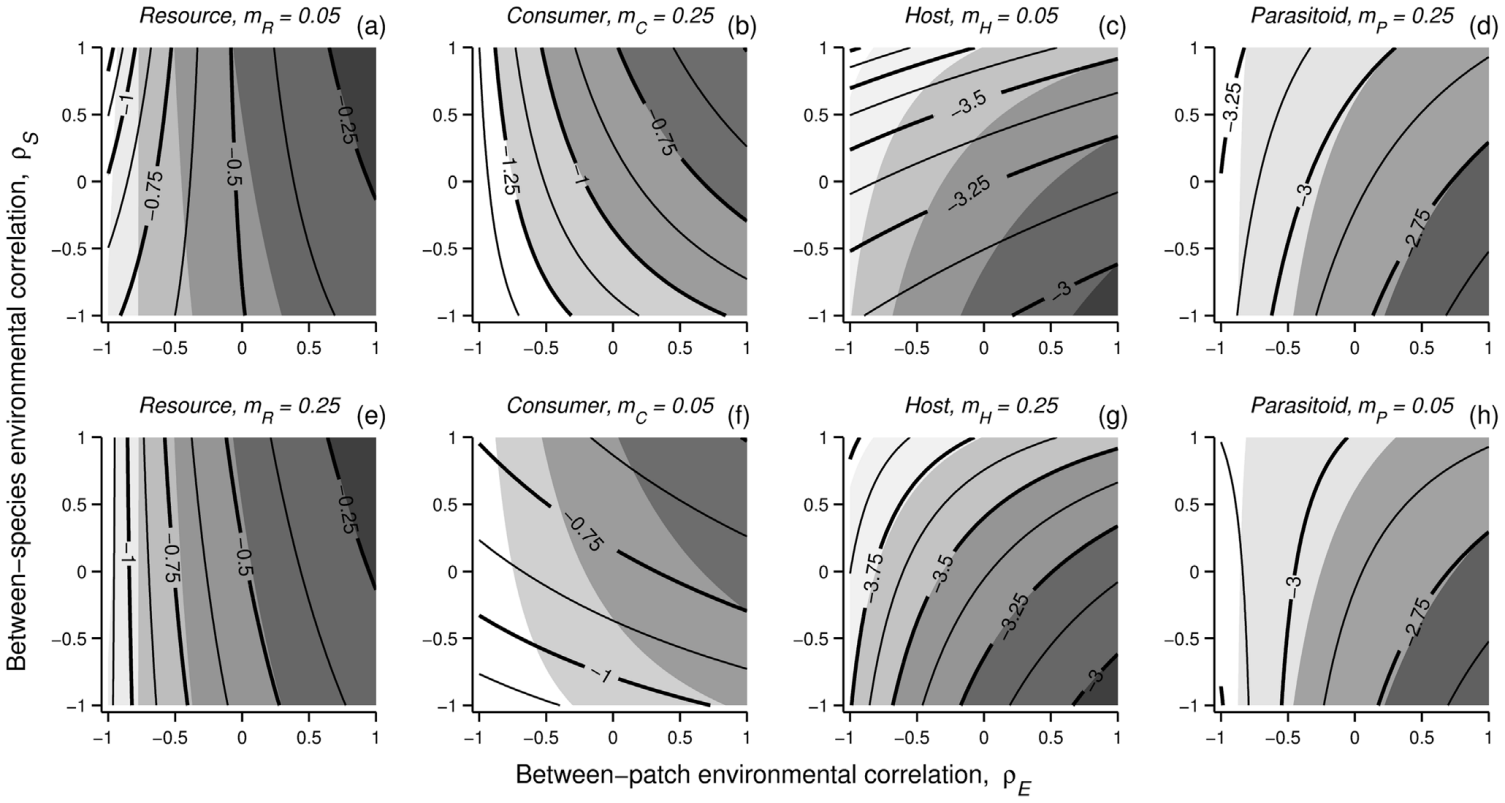
The effect of between-patch (*ρ_E_*) and between-species (*ρ_S_*) environmental correlation on population variability (log *CV*) in consumer–resource (a, b, e, f) and host–parasitoid (c, d, g, h) metacommunities with asymmetric dispersal between species. Contour lines represent (log) population *CV* under asymmetric dispersal, whereas the shading represents log *CV* under symmetric dispersal (with the same steps as the thick contour lines). Parameter values for *C–R* system: *r* = 1, *K* = 1, *a* = 2, *R*_0_ = 1.25, *e* = 0.5, *d* = 0.25. Parameter values for *H–P* system: *r* = 2, *q* = 0.5, *b* = 0.5. Environmental variation is serially uncorrelated white noise, with zero mean and variance σ^2^ = 0.01.

Local population variabilities under asymmetric dispersal (figs. 3,4) are qualitatively similar to those observed under symmetric dispersal (fig. 2). While the relative effect of *ρ_S_* depends on the between-species interaction, the relative effect of *ρ_E_* on each population depends on its dispersal capacity; limited dispersal weakens the direct influence of *ρ_E_* on the population variability of this species. Both of these factors affect the shape of the interaction between *ρ_E_* and *ρ_S_*. The interaction between *m_ik_* and *ρ_E_* is clearly seen in the competitive communities (fig. 3). While population variability at the species with a relatively high dispersal capacity (fig. 3b, d) remains practically the same as under symmetric dispersal (fig. 2a, b) (as the direct effect of *ρ_E_* remains the same), the effect of *ρ_E_* is weakened relative to *ρ_S_* at the species with limited dispersal (fig. 3a, c). The same applies also in the consumer–resource and host–parasitoid systems, when the consumer (fig. 4f) or the host (fig. 4c) is limited in its dispersal capacity.

The relationship between dispersal potential and *ρ_E_* does not hold in two special cases: under negative *ρ_E_*, when either (1) the resource (in a *C–R* system), or (2) the parasitoid (in a *H–P* system), is limited in its dispersal. (1) Under these conditions (*ρ_E_*<0), resource fluctuations are dampened by increasing *ρ_S_* (fig. 4a), despite of increased population synchrony. Under negative *ρ_S_* the *ρ_E_ρ_S_* term specifying the correlation between consumers and resource between patches is positive. Given that consumers are relatively well mixed between patches, high consumer dispersal from one patch coincides with high resource growth in the other patch (immigration and density are coupled between species), which amplifies resource fluctuations. The same mechanism applies in the *H*–*P* system (fig. 4h); reduced *ρ_S_* can dampen parasitoid fluctuations when the host is relatively well mixed as high host immigration is associated with high parasitoid growth rates.

## Discussion

While the results presented here might appear complex at first glance, they can in the end be understood by common underlying mechanisms. In these symmetric systems (patches are identical), common environmental forcing between patches synchronises population dynamics even in the absence of dispersal – the so-called ‘Moran effect’ [37]. When patches are connected by dispersal, decreasing the correlation between local environmental conditions (*ρ_E_*) dampens local population fluctuations due to a decoupling of immigration and population densities [9]. This stabilising effect of reduced *ρ_E_* is in agreement with previous studies on metapopulation dynamics [3], as well as spatially extended food webs [5], and experimental work on competitive metacommunities [6], [38]. The relative influence of *ρ_E_* on population variability depends on a species dispersal potential, which explains why species can differ in their responses to variation in *ρ_E_* under asymmetric dispersal.

Locally, between-species similarity in their responses to environmental fluctuations (*ρ_S_*) affects the synchrony among local populations. Increasing local synchrony can either amplify or dampen population fluctuations, depending on the interaction between species. In competitive communities synchronous dynamics are amplified by increased density dependence (overcompensatory dynamics) [12], [14], [15], [36]. A similar logic can be used to understand why the consumer – resources and host–parasitoid communities differ qualitatively in their responses to increasing *ρ_S_*. Instability in the *C–R* system is driven by overconsumption by the consumer (top-down control) [30], when resource growth is density-dependent. Here increasing *ρ_S_* promotes the consumer's coupling to the resource, which amplifies population fluctuations. This differs from the result by Vasseur and Fox [22], who reported that increasing *ρ_S_* between two intermediate consumers in a diamond-shaped food web reduces population variability, due cascading effects on the resource and the top predator.

Contrary to the *C–R* community, the run-away behaviour of the *H–P* model is driven by the host's lack of density-dependence (bottom-up effect). Increasing *ρ_S_* then promotes the parasitoids ability to track host growth, which reduces population variability. The importance of top-down control versus bottom-up effects in affecting the response of exploitative communities to forced synchronisation can be further highlighted by considering a host–parasitoid model with density-dependent host growth [25], [39]. In such a case, the *C–R* and *H–P* models produce qualitatively matching results considering the influence of increasing between-species environmental correlation (Figure S3).

### Interpreting environmental covariance

The between-patch environmental correlation (*ρ_E_*) could be interpreted as the degree of spatial autocorrelation, as this measure is likely to vary, e.g., due to increasing spatial distance between patches [4], [40]. Spatial autocorrelation in environmental conditions has been shown to play an important role, e.g., in population extinction risk [41]–[43]. In addition to spatial autocorrelation, temporal autocorrelation in local environmental conditions can also have important consequences for population extinction risk [2]. While this aspect was not considered here for simplicity, it is likely that temporal autocorrelation further affects the interaction between spatial correlation (*ρ_E_*) and between-species environmental correlation (*ρ_S_*), as it is known to interact with *ρ_S_* in driving population variability in isolated communities [12], [14], [15], and to interact with *ρ_E_* in competitive metacommunities in affecting population persistence [38]. Considering this alternative is beyond the scope of this paper, but the analytical methods used here are easily extended to account for temporally (serially) autocorrelated noise (Appendix S1) [12], [14], [15].

The magnitude of the between-species environmental correlation is likely to depend on external factors, namely the presence of other species in the community. Without any adjustment to species behaviour, varying community composition can affect patterns in *ρ_S_*(*i,j*) (the environmental correlation between a pair of species *i* and *j* in a community), if species respond differently to fluctuations in environmental conditions (such as temperature or precipitation). However, on top of these intrinsic differences in species environmental responses, the *ρ_S_*(*i,j*)'s can be further modified by individual behaviour. Interspecific competition can lead to changes in species resource utilization patterns [44]–[46] and if these resources are subject to environmental stochasticity, this can in turn result in variation in the *ρ_S_*(*i,j*)'s. The presence of predators can be associated with altered patterns in prey habitat use [47]. This can affect the way prey is influenced by environmental fluctuations, potentially altering the correlation between environmental responses of the predator and the prey.

### Extinction risk

In spatial systems, local population variability and global extinction risk are intimately related to patterns in population synchrony [3], [8], [10]. Extinction risk is generally increased by the Moran effect due to increased population synchrony [10]. Dispersal on the other hand can either synchronise or desynchronise local populations, depending on, e.g., spatial heterogeneity, local dynamics, and dispersal strategies [9], [48], [49].

Recently, Abbott [3] suggested that increasing population synchrony can either increase or decrease population variability, depending on the mechanism controlling the level of between-patch population synchrony; if synchrony varies due to population dispersal propensity or increased local growth rates, higher synchrony is stabilising, whereas other synchronising factors (such as increasing between-patch environmental correlation) tend to be destabilising. This is in agreement of the present results. However, a previously unconsidered aspect is that local between-species environmental correlation can also affect population synchrony between patches. This is simply a statistical consequence of *ρ_S_* affecting local population variances *V_ii_.* When the variances are increased, while between-population covariances *V_ij_* are unaffected, the between-species cross-correlation (*COR*(*X_i,_ X_j_*)) necessarily decreases (by definition).

### Dispersal asymmetry

Here I consider both symmetric and asymmetric dispersal between species, for completeness. Clearly, it is more realistic to assume that species differ in their dispersal potential. While predators are usually more mobile than their prey [50], many migratory species encounter sedentary predators in different habitats they visit [51]. Whether hosts or their parasitoids have higher dispersal capacities is likely to vary among taxa. For example, the Granville fritillary butterfly (*Melitea cinxia*) has two parasitoids that differ in their dispersal capacity in comparison with their host. *Cotesia melitaearum* is clearly less dispersive than the host, whereas *Hyposoter horticola* is at least as dispersive as the host [52].

For simplicity, species are here identical in the competitive communities. Thus, the identity of a dispersal-limited species is arbitrary. A logical outcome of dispersal limitation is that the relative influence of between-patch environmental correlation (*ρ_E_*) on population variance is reduced. In the exploitative communities this also applies to the species that has more control over the local dynamics; the consumer in the *C–R* system and the host in the *H–P* system. With the other species (resource or parasitoid) dispersal limitation can lead to a qualitative change in the effect of increasing *ρ_S_* under negative *ρ_E_*. In the *C–R* system, for example, this arises because the immigration of the consumer is coupled with resource density. This is inline with the observation that decoupling between immigration and density (within species) dampens population fluctuations [9]. These results also support the finding that the identity of a mobile species in a food web can be important for food web dynamics in metacommunities [53].

### Model assumptions and the robustness of results

I have here assumed that dispersal is a simple density independent reallocation of individuals [3], [25], [54]. It is, however, unlikely that individual dispersal strategies remain independent of their environments [55]–[60]. For example, spatial autocorrelation in environmental conditions can affect the evolution of dispersal modes [58], [60], such that higher spatial autocorrelation selects for longer dispersal distance [60]. While all dispersal propensities are equally favoured in perfectly correlated environments, increasingly negative between-patch environmental correlation selects for intermediate dispersal propensities [58]. The environmental correlation structure can also affect acquisition of information used to make dispersal decision, which could affect the evolution of dispersal strategies [56]. Thus, incorporating other dispersal strategies that depend on population densities, local environmental conditions, or their combination [49] is an import direction for future research.

The analytical predictions from figure 2 were tested using extensive numerical simulations (Text S1). These results (Figure S4) show good qualitative agreement with the analytical model (fig. 2). This also applies to asymmetric dispersal (not shown). However, as the models considered here (eqns. 2–4) are all non-linear, the accuracy of the linearization around the system equilibrium (eqn. 5) is only accurate when perturbations around this equilibrium are relatively small. Therefore, increasing the size of environmental fluctuations can potentially result in discrepancies between simulation and analytical results, as stronger forcing brings the system increasingly far away from the deterministic equilibrium [36]. As the environmental variance appears only as a linear scaling factor in eqn. (5), varying *σ* ^2^ has no qualitative effect on the analytical results.

An important assumption here is to only consider stable equilibrium dynamics. This is often assumed either to facilitate interpretation of resonance patterns between intrinsic dynamics and environmental forcing [36], [61], or to apply linearisation of the system to generate analytical results [12], [13]. However, non-equilibrium dynamics can have important consequences for spatial community dynamics under stochastic environments [5]. The influence of non-equilibrium dynamics was again tested with stochastic simulations, using the consumer–resource model with cyclic dynamics in the absence of stochasticity. The results from this simulation experiment (Figure S5) indicate that analytical results (fig. 2c, d) are not qualitatively sensitive to the assumption of stable equilibrium dynamics (at least under relatively weak noise). This is not very surprising, as the parameters used for stable dynamics are associated with dampened oscillations to equilibrium following perturbations. This means that under stochastic variation the system is constantly undergoing cyclic, transient dynamics, which is evident from figure 1c.

### Implications

The analysis presented here makes predictions about the behaviour of extremely simple systems under stochastic environmental variation. These predictions, at least those considering symmetric dispersal, could be tested using existing procedures for experimental microcosms [38], [62]–[64]. Between-patch environmental correlation would then be manipulated by simply tuning the correlation in, e.g., temperature [65] or light spectrum [64], between ‘patches’. Adjusting between-species environmental correlation would in turn require selecting a pair of species with desired differences in their adaptation along the focal environmental variable.

Predictions about the dynamics of populations and communities under spatio-temporal environmental variation are needed, e.g., for mitigation of climatic perturbations and planning sustainable population management across spatial landscapes. The present analysis is the first step towards a more general understanding of how metacommunities behave in stochastic environments. Considering the effect of spatial heterogeneity in patch quality [29], [63], [66]–[68] in association with the environmental covariance structure is an interesting direction for future research. A recent study demonstrated how spatial heterogeneity and spatially restricted harvesting can generate ecological traps, leading to global population extinction [69]. Although some work has been done on how exploitation interacts with spatial population processes in stochastic environments [8], more work is needed to better understand potential interactions between spatio-temporal environmental fluctuations and exploitation in spatial systems with between-patch dispersal. The works by Gouhier et al. [70] – showing that localised dispersal can generate non-stationary spatio-temporal patterns in population abundances, and Blowes and Connoly [40] – showing that the decay of between-patch environmental correlation with between-patch spatial distance can affect metapopulation persistence, make a promising step in this direction, both having direct implications to effective design of reserve networks.

## Supporting Information

Figure S1 **The dependency of the combined term** ***ρ_E_ρ_S_*** **in the environmental covariance matrix C on its components** ***ρ_E_*** **(between-patch environmental correlation) and** ***ρ_E_*** **(between-species environmental correlation), see eqn. (A.3) in Appendix S1.**
(PDF)

Figure S2 **Between–population synchrony in simple two-species two-patch metacommunities depends on an interaction between the environmental correlation between patches (** ***ρ_E_*** **) and the local environmental correlation between species (** ***ρ_S_*** **).** In competitive communities species intrinsic dynamics are either (a) undercompensatory or (b) overcompensatory. In exploitative communities the interaction involves either (c, d) consumer–resource dynamics, or (e, f) host–parasitoid dynamics. The contours represent analytical approximations of the between–patch population cross-correlation for each species. Results are based on an intermediate level of symmetric dispersal for both species, *m_ik_*  =  *m*  = 0.25. Parameters: (a) *r* = 1, *θ* = 0.25, α = 0.5; (b) *r* = 1, *θ* = 1.25, α = 0.5; (c, d) *r* = 1, *K* = 1, *a* = 2, *R*_0_ = 1.25, *e* = 0.5, *d* = 0.25; (e, f) *r* = 2, *q* = 0.5, *b* = 0.5. Environmental variation is serially uncorrelated white noise, with zero mean and variance σ^2^ = 0.01.
(PDF)

Figure S3
**Population variability (log** ***CV*** **), affected by between–patch (** ***ρ_E_*** **) and between–species environmental correlation (** ***ρ_S_*** **), in a host–parasitoid metacommunity.** The local community dynamics are modelled as (Beddington et al., 1975; Ranta et al., 2008): 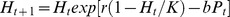, 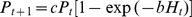, where *r* and *K* are the intrinsic growth rate and carrying capacity of the host (*H*), and *b* and *c* are the attack rate and conversion efficiency of the parasitoid (*P*). Parameters: *r* = 1, *K* = 1, *c* = 2, *b* = 1, *m_ik_*  =  *m* = 0.25. Environmental variation is white noise (zero mean and variance σ^2^ = 0.01).(PDF)

Figure S4 **Analytically derived population variabilities (log** ***CV*** **; red contour lines) match qualitatively with those resulting from stochastic simulations (black contour lines and shading).** In competitive communities species intrinsic dynamics are either (a) undercompensatory or (b) overcompensatory. In exploitative communities the interaction involves either (c, d) consumer–resource dynamics, or (e, f) host–parasitoid dynamics. The **black** contours represent the logarithm of simulated population *CV*, based on 100 independent replicates. Results are based on an intermediate level of symmetric dispersal for both species, *m_ik_*  =  *m* = 0.25. Parameters: (a) *r* = 1, *θ* = 0.25, α = 0.5; (b) *r* = 1, *θ* = 1.25, α = 0.5; (c, d) *r* = 1, *K* = 1, *a* = 2, *R*_0_ = 1.25, *e* = 0.5, *d* = 0.25; (e, f) *r* = 2, *q* = 0.5, *b* = 0.5. Environmental variation is serially uncorrelated white noise, with zero mean and variance σ^2^ = 0.01, for both analytical and simulation results. Simulation-based *CV* –values have been scaled arbitrarily to better coincide with the corresponding analytically derived values.
(PDF)

Figure S5 **Population variability (log** ***CV*** **), affected by between–patch (** ***ρ_E_*** **) and between–species environmental correlation (** ***ρ_S_*** **), in a consumer–resource metacommunity with cyclic local dynamics.** Parameters: *r* = 1, *K* = 1, *a* = 2, *R*_0_ = 1.25, *e* = 0.5, *d* = 0.1, *m_ik_*  =  *m* = 0.25. The data (shaded contours) represents means over 100 replicates, while the contour lines give a smoothing of the original data. Environmental variation is serially uncorrelated white noise, with zero mean and variance σ^2^ = 0.01.
(PDF)

Appendix S1 **Details on analytical treatment of environmental noise and population variance.**
(DOCX)

Text S1 **Additional information on the environmental correlation structure, simulation methods, and additional results.**
(DOCX)
