# Correction: Spatio-Temporal Environmental Correlation and Population Variability in Simple Metacommunities

**DOI:** 10.1371/annotation/5773bccc-fe71-4924-bd35-8b7ac7d23d08

**Published:** 2013-11-01

**Authors:** Lasse Ruokolainen

Errors were introduced to some equations during the production process. In equation (1a) in function f(), the subscript for the first term within the brackets should be 1k,t, not ik,t. Please view the correct equation here: 


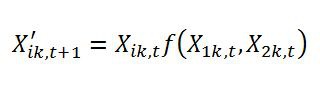


In equation (3b), within the square brackets, the nominator of the first term is missing parameter 'e'. Please view the correct equation here: 


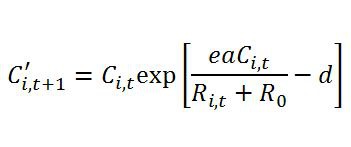


In the final two sentences of the paragraph after equations 3a and 3b, all instances of B0 should be replaced with R0. Those sentences should read: "For the consumer to persist, it is required that d < Kae/(R0 + K). A general requirement for the stability of this equilibrium is thatd/e > a(R0 – K)/(R0 + K)" 

