# Supplementary figures and images for: Spatio-Temporal Environmental Correlation and Population Variability in Simple Metacommunities
Source: PLoS One. 2013 Aug 30;8(8):e72325. doi: 10.1371/journal.pone.0072325 (PMC3758301; doi:10.1371/journal.pone.0072325)

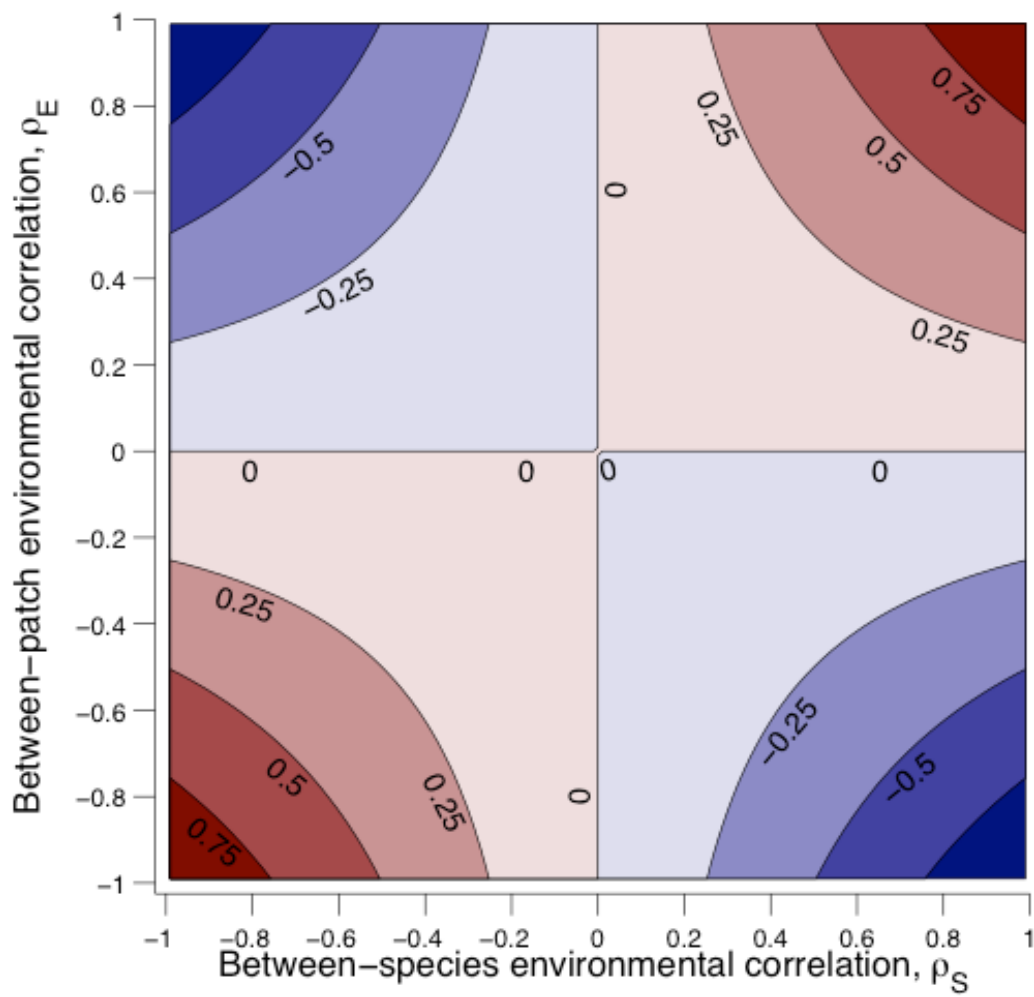

Supplement: Figure S1 — The dependency of the combined term ρEρS in the environmental covariance matrix C on its components ρE (between-patch environmental correlation) and ρE (between-species environmental correlation), see eqn. (A.3) in Appendix S1. (PDF) [file pone.0072325.s001.pdf]

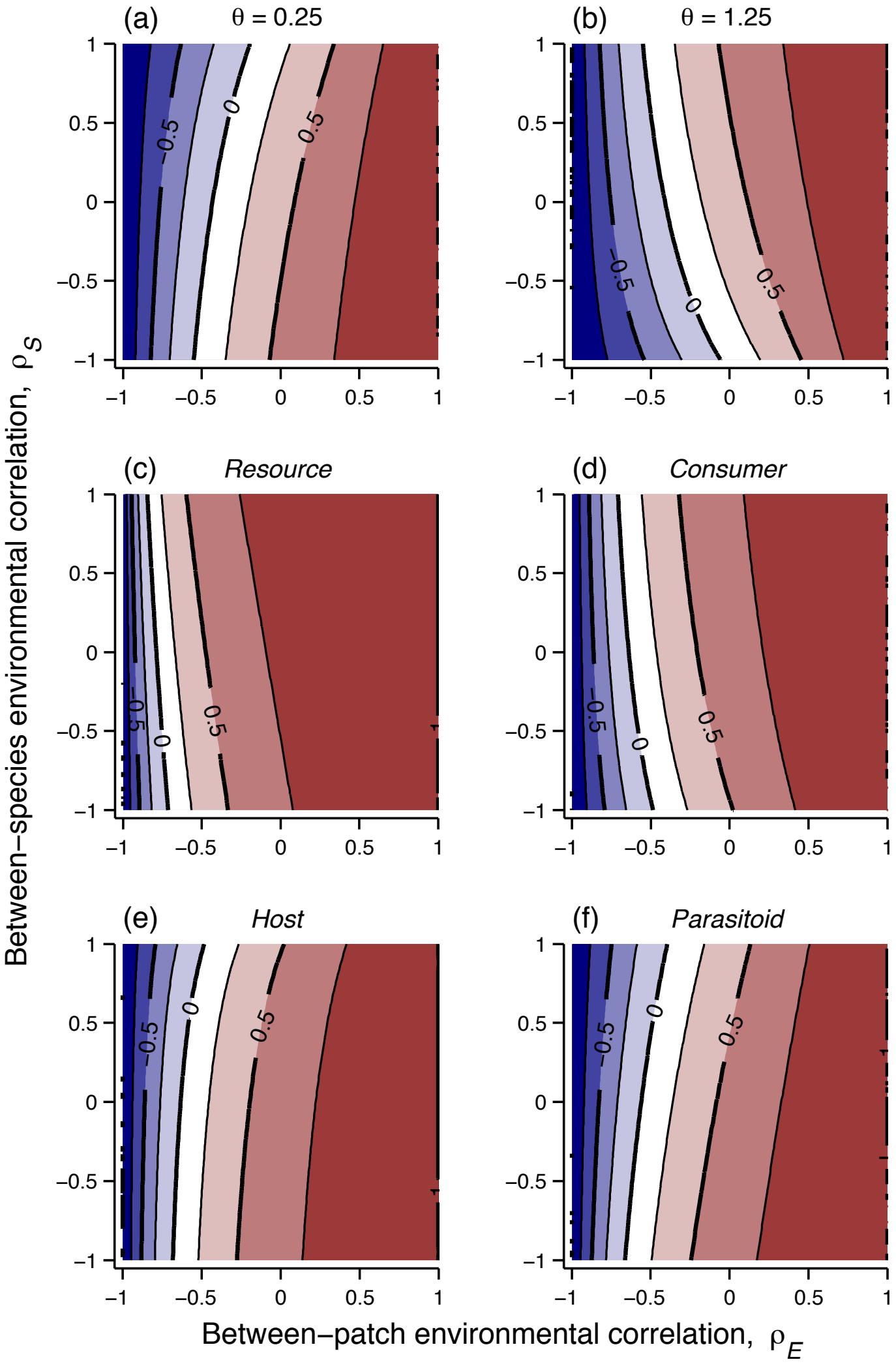

Supplement: Figure S2 — Between–population synchrony in simple two-species two-patch metacommunities depends on an interaction between the environmental correlation between patches ( ρE ) and the local environmental correlation between species ( ρS ). In competitive communities species intrinsic dynamics are either (a) undercompensatory or (b) overcompensatory. In exploitative communities the interaction involves either (c, d) consumer–resource dynamics, or (e, f) host–parasitoid dynamics. The contours represent analytical approximations of the between–patch population cross-correlation for each species. Results are based on an intermediate level of symmetric dispersal for both species, mik = m = 0.25. Parameters: (a) r = 1, θ = 0.25, α = 0.5; (b) r = 1, θ = 1.25, α = 0.5; (c, d) r = 1, K = 1, a = 2, R 0 = 1.25, e = 0.5, d = 0.25; (e, f) r = 2, q = 0.5, b = 0.5. Environmental variation is serially uncorrelated white noise, with zero mean and variance σ2 = 0.01. (PDF) [file pone.0072325.s002.pdf]

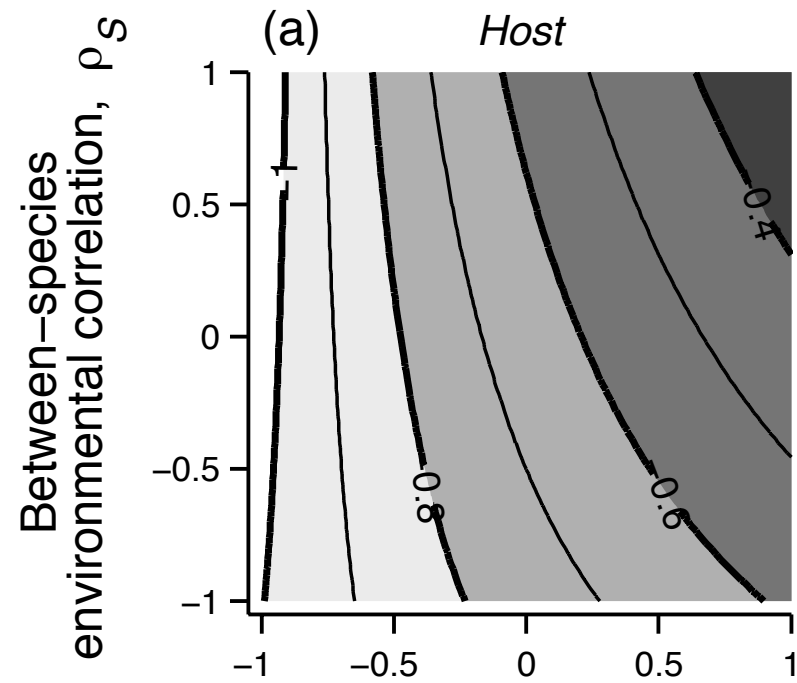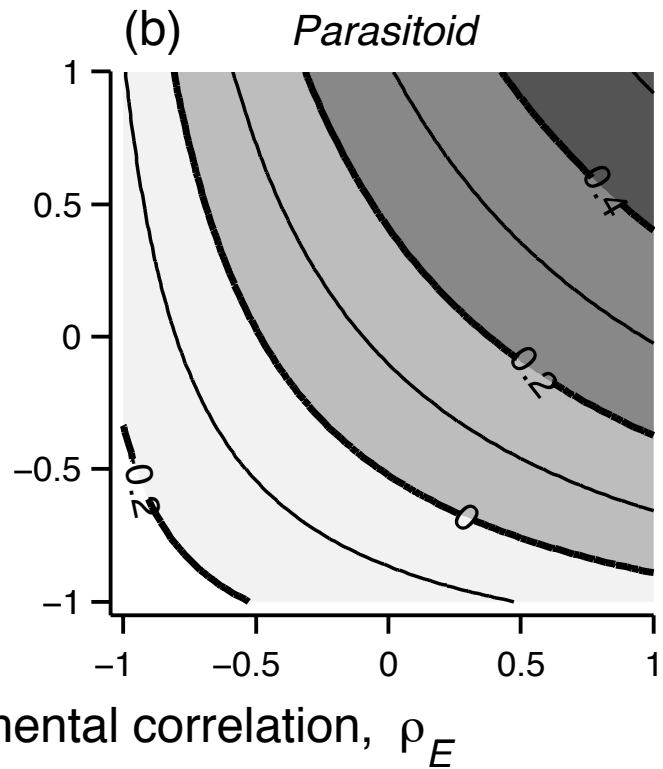

Supplement: Figure S3 — Population variability (log CV ), affected by between–patch ( ρE ) and between–species environmental correlation ( ρS ), in a host–parasitoid metacommunity. The local community dynamics are modelled as (Beddington et al., 1975; Ranta et al., 2008): , , where r and K are the intrinsic growth rate and carrying capacity of the host (H), and b and c are the attack rate and conversion efficiency of the parasitoid (P). Parameters: r = 1, K = 1, c = 2, b = 1, mik = m = 0.25. Environmental variation is white noise (zero mean and variance σ2 = 0.01). (PDF) [file pone.0072325.s003.pdf]

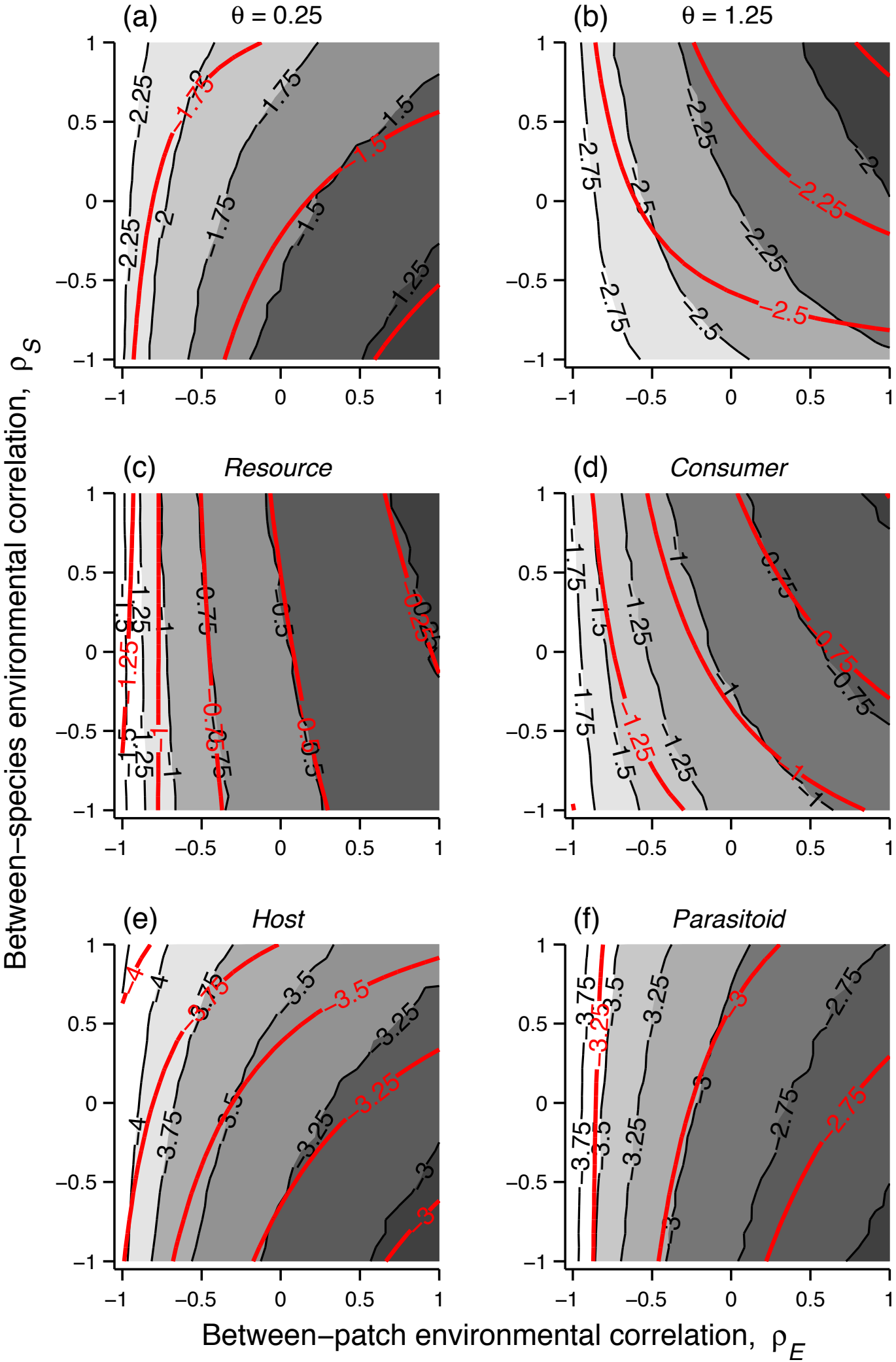

Supplement: Figure S4 — Analytically derived population variabilities (log CV ; red contour lines) match qualitatively with those resulting from stochastic simulations (black contour lines and shading). In competitive communities species intrinsic dynamics are either (a) undercompensatory or (b) overcompensatory. In exploitative communities the interaction involves either (c, d) consumer–resource dynamics, or (e, f) host–parasitoid dynamics. The black contours represent the logarithm of simulated population CV, based on 100 independent replicates. Results are based on an intermediate level of symmetric dispersal for both species, mik = m = 0.25. Parameters: (a) r = 1, θ = 0.25, α = 0.5; (b) r = 1, θ = 1.25, α = 0.5; (c, d) r = 1, K = 1, a = 2, R 0 = 1.25, e = 0.5, d = 0.25; (e, f) r = 2, q = 0.5, b = 0.5. Environmental variation is serially uncorrelated white noise, with zero mean and variance σ2 = 0.01, for both analytical and simulation results. Simulation-based CV –values have been scaled arbitrarily to better coincide with the corresponding analytically derived values. (PDF) [file pone.0072325.s004.pdf]

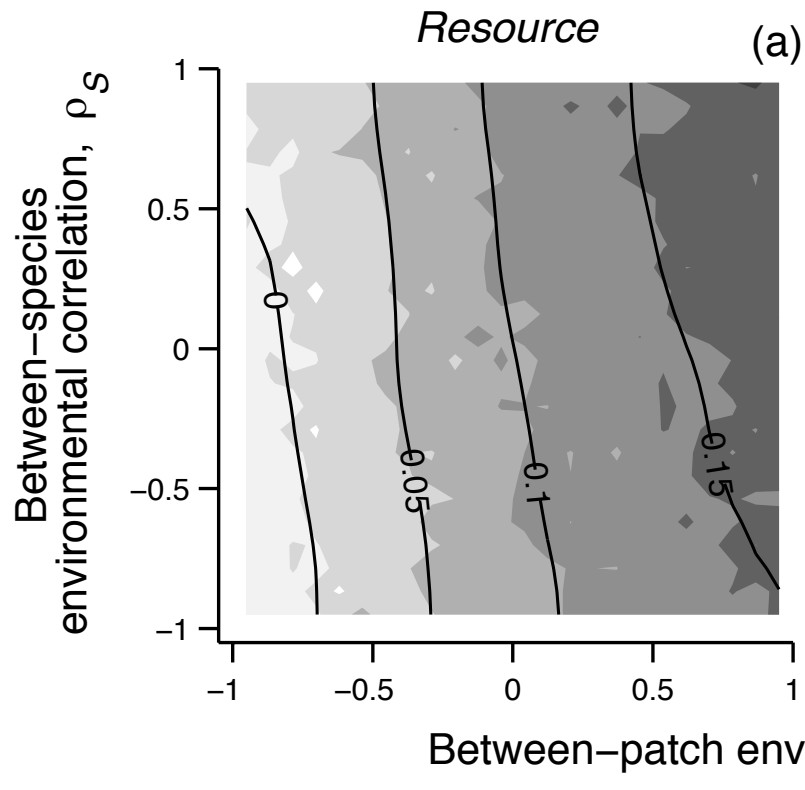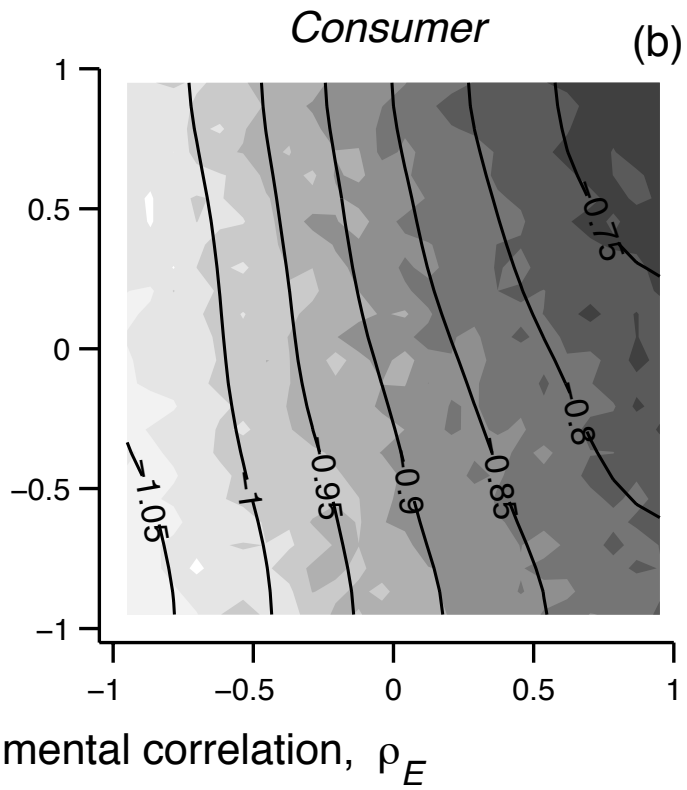

Supplement: Figure S5 — Population variability (log CV ), affected by between–patch ( ρE ) and between–species environmental correlation ( ρS ), in a consumer–resource metacommunity with cyclic local dynamics. Parameters: r = 1, K = 1, a = 2, R 0 = 1.25, e = 0.5, d = 0.1, mik = m = 0.25. The data (shaded contours) represents means over 100 replicates, while the contour lines give a smoothing of the original data. Environmental variation is serially uncorrelated white noise, with zero mean and variance σ2 = 0.01. (PDF) [file pone.0072325.s005.pdf]
