# Supplementary material for: Spatio-Temporal Environmental Correlation and Population Variability in Simple Metacommunities
Source: PLoS One. 2013 Aug 30;8(8):e72325. doi: 10.1371/journal.pone.0072325 (PMC3758301; doi:10.1371/journal.pone.0072325)
Supplement: Appendix S1 — Details on analytical treatment of environmental noise and population variance. (DOCX) [file pone.0072325.s006.docx]

# Appendix S1

## Environmental noise

The focus here is in analysing the influence of spatio-temporal covariance in species-specific responses to environmental fluctuations on population variances. A given environmental correlation structure combining between-patch environmental similarity *ρ_E_* and within-patch between-species environmental correlation *ρ_S_* is found by , where • indicates an element-wise product of matrices. Matrices **D** and **R** in the exponents of *ρ_E_* and *ρ_S_* contain the between-patch distances (0 = same patch, 1 = different patch) and between-species relatedness (0 = same species, 1 = different species), respectively:

 , (A.1)

 , (A.2)

Then, matrix **C** containing between-species environmental covariances is found as:

 , (A.3)

where σ^2^ is the environmental variance. The exponentiation of *ρ_E_* and *ρ_S_* with **D** an **R**, respectively, is made element-wise. As a result, zeros in matrices **D** and **R** become replaced with ones, whereas the ones are replaced by *ρ_E_* and *ρ_S_*, respectively. The resulting covariance matrix **C** is positive definite for all combinations of *ρ_E_* and *ρ_S_* (which is required for the covariance structure to be feasible), as long as |*ρ_E_*, *ρ_S_* | < 1. The combined term *ρ_E_ρ_S_* describing the environmental correlation between species between patches, which dependence on *ρ_E_* and *ρ_S_* is illustrated in Figure S1.

## Population variance

For analytically deriving within-patch population variances, several matrices controlling different ecological processes, need to be defines. For describing between-patch dispersal, a dispersal matrix is needed:

$\mathbf{M}=\left[ \begin{matrix} (1-m_{11}) & 0 & m_{12} & 0 \\ 0 & (1-m_{21}) & 0 & m_{22} \\ m_{11} & 0 & (1-m_{12}) & 0 \\ 0 & m_{21} & 0 & (1-m_{22}) \end{matrix} \right]$ , (A.4)

This dispersal matrix can be combined with the Jacobian matrix, describing local dynamics, to form a metacommunity matrix:

$\mathbf{B}=\mathbf{MJ}$ , (A.5)

where **J** is the Jacobian matrix of the local communities:

$\mathbf{J}=\left[ \begin{matrix} \mathbf{J}_{1} & \mathbf{0} \\ \mathbf{0} & \mathbf{J}_{2} \end{matrix} \right]$ . (A.6)

Here **J***_i_* is the jacobian for local community *i* and **0** is a matrix of zeros of the same size as **J***_i_* (here 2 x 2 in size).

Assuming that patches are identical (population parameters are independent of spatial location), species equilibrium densities become independent of the movement parameter *m_ik_.* This simplification makes it possible to find the metacommunity matrices for each system. The local Jacobian matrices **J***_i_* are defined as follows.

Competition model:

$\mathbf{J}_{i}=\left[ \begin{matrix} 1-\frac{\theta r}{\alpha+1} & -\frac{\alpha\theta r}{\alpha+1} \\ -\frac{\alpha\theta r}{\alpha+1} & 1-\frac{\theta r}{\alpha+1} \end{matrix} \right]$ , (A.7)

consumer–resource model:

$\mathbf{J}_{i}\mathbf{=}\left[ \begin{matrix} 1+\frac{dr\left( ea\left( R_{0}-K \right)+R_{0}d+Kd \right)}{Kae\left( d-ae \right)} & -de \\ er-\frac{dr\left( R_{0}+K \right)}{Ka} & 1 \end{matrix} \right]$ **, (A.8)**

host–parasitoid model:

$\mathbf{J}_{i}\mathbf{=}\left[ \begin{matrix} 1 & \frac{qr\left( r^{-1/q}-1 \right)}{r-1} \\ 1-\frac{1}{r} & \boldsymbol{-}\frac{q\left( r^{-1/q}-1 \right)}{r-1} \end{matrix} \right]$ **. (A.9)**

In the absence of environmental autocorrelation (κ = 0) population variances can be found analytically by first transforming system dynamics to run along the eigenvectors of **B** (Ripa and Ives, 2003). For this a matrix **T**, with each column representing an eigenvectors of **B**, as well as the eigenvalues *λ_i_* of **B**. The variance-covariance matrix in the eigenvector space **Υ** is then found as (Ripa and Ives, 2003):

$\nu_{ij}=\frac{\gamma_{ij}}{1-\lambda_{i}\lambda_{j}}$ , (A.10)

where *γ_ij_* indicate the elements of the transformed environmental covariance matrix **C**: **Γ** = **T**^–1^**C**(**T**^–1^)*^T^*. Population variances can be obtained by a back-transformation from eigenvector space to population coordinates, **V** = **TΥT**^–1^, where **V** is the population variance-covariance matrix. Using this method population variance in the competitive metacommunity becomes:

$V=\frac{\sigma^{2}}{\Theta^{2}}\left[ \frac{(\rho_{E}+1)(\rho_{S}-1)}{\lambda_{1}^{2}-1}-\frac{\left( \rho_{E}+1 \right)\left( \rho_{S}+1 \right)}{\lambda_{2}^{2}-1}-\frac{\left( \rho_{E}-1 \right)\left( \rho_{S}-1 \right)}{\lambda_{3}^{2}-1}+\frac{(\rho_{E}-1)(\rho_{S}+1)}{\lambda_{4}^{2}-1} \right]$ , (A.11)

where *σ* is the SD of environmental noise. Under symmetric dispersal (*m_ik_* = *m*) the Jacobian eigenvalues are: *λ*_1_ = (*α* – *θr* + *αθr* + 1)/(*α* + 1), *λ*_2_ = 1 – *θr*, *λ*_3_ = [*α*(*θr* + 1) – 2*m*(*θr* – *αθr* – *α* – 1) – *θr* + 1]/ (*α* + 1), *λ*_4_ = 2*m*(*θr* – 1) – *θr* + 1. Parameter Θ is the order of matrix **B** (here Θ = 4). Eqn. (A.11) is actually just the sum of the variances along the Jacobian eigenvectors divided by the number of eigenvectors, i.e., a mean over the variances in eigenvector space.

Ideally, it should be possible to partition population variance into components depending on *ρ_E_* and *ρ_S_* alone, and their interaction *ρ_E_ρ_S_*. In the absence of the interaction term the independent components are easily obtained, by setting *α* = 0 or *m* = 0, respectively:

$\left. V \right|_{\alpha=0}=\frac{\sigma^{2}}{\Theta}\left( \frac{\rho_{E}-1}{\lambda_{1}^{2}-1}-\frac{\rho_{E}+1}{\lambda_{2}^{2}-1} \right)$ (A.12a)$\left. V \right|_{m=0}=\frac{\sigma^{2}}{\Theta}\left( \frac{\rho_{S}-1}{\lambda_{4}^{2}-1}-\frac{\rho_{S}+1}{\lambda_{2}^{2}-1} \right)$ . (A.12b)

Eqn. (A.11) can be rearranged to display the interrelation between the Jacobian eigenvalues, and *ρ_E_*, *ρ_S_* and *ρ_E_ρ_S_*, including a term that is independent of the environmental correlation structure:

$V=\frac{\sigma^{2}\rho_{E}}{\Theta^{2}}\left( \frac{1}{\lambda_{3}^{2}-1}+\frac{1}{\lambda_{4}^{2}-1}-\frac{1}{\lambda_{1}^{2}-1}-\frac{1}{\lambda_{2}^{2}-1} \right)+\frac{\sigma^{2}\rho_{S}}{\Theta^{2}}\left( \frac{1}{\lambda_{1}^{2}-1}+\frac{1}{\lambda_{3}^{2}-1}-\frac{1}{\lambda_{2}^{2}-1}-\frac{1}{\lambda_{4}^{2}-1} \right)$ $+\frac{\sigma^{2}\rho_{E}\rho_{S}}{\Theta^{2}}\left( \frac{1}{\lambda_{1}^{2}-1}+\frac{1}{\lambda_{4}^{2}-1}-\frac{1}{\lambda_{2}^{2}-1}-\frac{1}{\lambda_{3}^{2}-1} \right)-\frac{\sigma^{2}}{\Theta^{2}}\sum_{i=1}^{4} \frac{1}{\lambda_{i}^{2}-1}$ . (A.13)

Here it becomes more clear that the effect of *ρ_E_* depends on the relative strength of dispersal mediated processes (*λ*_3_ and *λ*_4_) and local processes (*λ*_1_ and *λ*_2_), whereas the effect of *ρ_S_* depends on the relative strength of interspecific interaction (*λ*_1_ and *λ*_3_) and intrinsic density regulation (*λ*_2_ and *λ*_4_). Finally, how the magnitude of the interaction term *ρ_E_ρ_S_* depends on different processes is not that clear. The magnitude of the eigenvalue-sum that multiplies the *ρ_E_ρ_S_* term increase with increasing *θ* and *r*, decreases with α, and decreases (increases) with *m* when intrinsic dynamics are undercompensatory (overcompensatory).

An analytical expression for population variance in autocorrelated environments is also possible to derive (Ripa and Ives, 2003; Ruokolainen and Ripa, 2012):

$V=\frac{\sigma^{2}}{\Theta^{2}}\left[ \frac{\left( \lambda_{2}\kappa+1 \right)\left( \rho_{E}+1 \right)\left( \rho_{S}+1 \right)}{\left( \lambda_{2}^{2}-1 \right)\left( \lambda_{2}\kappa-1 \right)}-\frac{\left( \lambda_{1}\kappa+1 \right)\left( \rho_{E}+1 \right)\left( \rho_{S}-1 \right)}{\left( \lambda_{1}^{2}-1 \right)\left( \lambda_{1}\kappa-1 \right)} \right.$ $\left. -\frac{(\lambda_{3}\kappa+1)(\rho_{E}-1)(\rho_{S}-1)}{(\lambda_{3}^{2}-1)(\lambda_{3}\kappa-1)}+\frac{(\lambda_{4}\kappa+1)(\rho_{E}-1)(\rho_{S}+1)}{(\lambda_{4}^{2}-1)(\lambda_{4}\kappa-1)} \right]$ , (A.14)

where κ is the environmental autocorrelation. Systems with potentially complex eigenvalues and eigenvectors, such as eqns. A.8 and A.9, are harder to solve (Ripa and Ives, 2003). The solutions (if even obtainable) are very complex (due to the Jacobian eigenvectors being dependent on model parameters), so they will not be treated here.

**Supplementary References**

Ripa, J., Ives, A.R., 2003. Food web dynamics in correlated and autocorrelated environments. Theor. Popul. Biol. 64, 369–384.

Ruokolainen, L., Ripa, J., 2012. The strength of species interactions modifies population responses to environmental variation in competitive communities. J. Theor. Biol. 310, 199–205.
