# Supplementary material for: Spatio-Temporal Environmental Correlation and Population Variability in Simple Metacommunities
Source: PLoS One. 2013 Aug 30;8(8):e72325. doi: 10.1371/journal.pone.0072325 (PMC3758301; doi:10.1371/journal.pone.0072325)
Supplement: Text S1 — Additional information on the environmental correlation structure, simulation methods, and additional results. (DOCX) [file pone.0072325.s007.docx]

# Text S1

## Environmental correlation structure

In the Appendix in the main text I describe a method for deriving the environmental correlation matrix **C**. Within this matrix, specific cells contain a hybrid term of between-patch environmental correlation (*ρ_E_*) and between–species environmental correlation (*ρ_E_*), *ρ_E_ ρ_S_*. The dependency of this term on its components is depicted in Figure S1.

## Simulation

I used stochastic simulations to confirm the analytical results presented in the main text. These simulations were performed as follows. For the present exercise four time series are needed (species-specific noise for two species in two patches), but the method described below can be used to compose any number of inter-correlated time series. First a covariance matrix **C** is constructed to specify how species- and patch-specific environmental effects are correlated with each other (see Appendix in main text). Matrix **C** contains within-patch environmental correlation between species (*ρ_S_*), between-patch environmental correlation for each species (*ρ_E_*), and their combination (*ρ_S_ρ_E_*) telling how well the environmental response of one species in one patch resembles that of the other species in the other patch.

To generate the environmental time series matrix **C** is first transformed using Cholesky decomposition (using the *chol* function in Matlab). The resulting matrix **A** is then combined with a matrix **X** of size *t_MAX_* times *n* (*n* = the number of populations x the number of patches), containing i.i.d. normal random variables, with zero mean and variance σ^2^, to get a multivariate AR(1) process:

$\mathbf{E}_{t}\boldsymbol{=}\kappa\boldsymbol{E}_{t-1}\boldsymbol{+}\mathbf{A}\mathbf{X}_{t-1}$ , (5)

where vector **E***_t_* contains environmental conditions for each population in each patch at time *t*. Parameter κ defines the first-order autocorrelation of the resulting environmental time series (Ripa and Lundberg, 1996). When κ < 0 the ‘blue’ environment is characterised by fast, high-frequency fluctuations, whereas under κ > 0 ‘red’ environmental fluctuations are dominated by slow, low-frequency fluctuations. In ‘white‘ noise (κ = 0) all frequencies have equal weight and thus fluctuations are completely random.

For each parameter combination metacommunity dynamics are simulated for *t_MAX_* = 25000 time steps (population densities initiated at random densities between [0,1]). The first 5000 time steps are discarded before further analysis. The data is used to calculated mean population variability for each species as the coefficient of variation [*CV_i_* = Σσ(*X_ik_*)/2*μ*(*X_ik_*)]. Each parameter combination is replicated 100 times. The results from these simulations, showing patterns in population *CV*’s, are presented in Figure S2, which shows good qualitative agreement between simulation and analytical results. This analysis considers only symmetric dispersal, but the same conclusion can be found for asymmetric dispersal.

## Extension to analytical results

In addition to population *CV,* I also calculated results for between–population synchrony between patches. Patterns in population variability (Fig. 3 in main text) are largely reflected in those in between–population synchrony (Figure S2). In the main text host–parasitoid dynamics are modelled using the so called negative-binomial model (Abbott, 2011). For comparison, I also investigated another model, with density dependent host growth (Beddington et al., 1975; Ranta et al., 2008). The resulting patterns in population variability are plotted in Figure S4.

**Supplementary References**

Abbott, K.C., 2011. A dispersal-induced paradox: synchrony and stability in stochastic metapopulations. Ecol. Lett. 14, 1158–1169.

Beddington, J.R., Free, C.A., Lawton, J.H., 1975. Dynamic complexity in predator-prey models framed in difference equations. Nature 255, 58–60.

McCann, K.S., Rooney, N., 2009. The more food webs change, the more they stay the same. Phil. Trans. R. Soc. B 364, 1789–1801.

Ranta, E., Fowler, M.S., Kaitala, V., 2008. Population synchrony in small-world networks. Proc R. Soc. B 275, 435–442.

Ripa, J., Lundberg, P., 1996. Noise colour and the risk of population extinctions. Proc R. Soc. Lond. B 263, 1751–1753.
